# Automatic Characterization of Prostate Suspect Lesions on T2-Weighted Image Acquisitions Using Texture Features and Machine-Learning Methods: A Pilot Study

**DOI:** 10.3390/diagnostics15010106

**Published:** 2025-01-04

**Authors:** Teodora Telecan, Cosmin Caraiani, Bianca Boca, Roxana Sipos-Lascu, Laura Diosan, Zoltan Balint, Raluca Maria Hendea, Iulia Andras, Nicolae Crisan, Monica Lupsor-Platon

**Affiliations:** 1 Department of Anatomy and Embryology, “Iuliu Hatieganu” University of Medicine and Pharmacy, 400012 Cluj-Napoca, Romania; t.telecan@gmail.com; 2 Department of Pathology, Country Emergency Clinical Hospital, 400347 Cluj-Napoca, Romania; 3 Department of Medical Imaging, “Iuliu Hatieganu” University of Medicine and Pharmacy, 400012 Cluj-Napoca, Romania; ccaraiani@yahoo.com (C.C.); bianca.petresc@gmail.com (B.B.); monica.lupsor@umfcluj.ro (M.L.-P.); 4 Department of Radiology, County Emergency Clinical Hospital, 400006 Cluj-Napoca, Romania; 5 Department of Radiology, “George Emil Palade” University of Medicine, Pharmacy, Science and Technology, 500139 Târgu Mureș, Romania; 6 Department of Computer Science, Faculty of Mathematics and Computer Science, “Babes-Bolyai” University, 400157 Cluj-Napoca, Romania; laura.diosan@ubbcluj.ro; 7 Department of Biomedical Physics, Faculty of Physics, “Babes-Bolyai” University, 400084 Cluj-Napoca, Romania; zoltan.balint@ubbcluj.ro; 8 Department of Pathology, “Iuliu Hatieganu” University of Medicine and Pharmacy, 400012 Cluj-Napoca, Romania; maria.bungardean@yahoo.com; 9 Department of Urology, “Iuliu Hatieganu” University of Medicine and Pharmacy, 400012 Cluj-Napoca, Romania; dr.iuliaandras@gmail.com (I.A.); drnicolaecrisan@gmail.com (N.C.); 10 Department of Urology, Clinical Municipal Hospital, 400139 Cluj-Napoca, Romania; 11 Department of Medical Imaging, Regional Institute of Gastroenterology and Hepatology “Prof. Dr. Octavian Fodor”, 400162 Cluj-Napoca, Romania

**Keywords:** radiomics, textural analysis, prostate cancer, mpMRI, artificial intelligence, machine learning, radical prostatectomy

## Abstract

**Background**: Prostate cancer (PCa) is the most frequent neoplasia in the male population. According to the International Society of Urological Pathology (ISUP), PCa can be divided into two major groups, based on their prognosis and treatment options. Multiparametric magnetic resonance imaging (mpMRI) holds a central role in PCa assessment; however, it does not have a one-to-one correspondence with the histopathological grading of tumors. Recently, artificial intelligence (AI)-based algorithms and textural analysis, a subdivision of radiomics, have shown potential in bridging this gap. **Objectives**: We aimed to develop a machine-learning algorithm that predicts the ISUP grade of manually contoured prostate nodules on T2-weighted images and classifies them into clinically significant and indolent ones. **Materials and Methods:** We included 55 patients with 76 lesions. All patients were examined on the same 1.5 Tesla mpMRI scanner. Each nodule was manually segmented using the open-source 3D Slicer platform, and textural features were extracted using the PyRadiomics (version 3.0.1) library. The software was based on machine-learning classifiers. The accuracy was calculated based on precision, recall, and F1 scores. **Results**: The median age of the study group was 64 years (IQR 61–68), and the mean PSA value was 11.14 ng/mL. A total of 85.52% of the nodules were graded PI-RADS 4 or higher. Overall, the algorithm classified indolent and clinically significant PCas with an accuracy of 87.2%. Further, when trained to differentiate each ISUP group, the accuracy was 80.3%. **Conclusions**: We developed an AI-based decision-support system that accurately differentiates between the two PCa prognostic groups using only T2 MRI acquisitions by employing radiomics with a robust machine-learning architecture.

## 1. Introduction

Prostate cancer (PCa) is the most frequent neoplasia in the male population [1], with more than 1.4 million new cases being diagnosed worldwide yearly [2]. According to the International Society of Urological Pathology (ISUP), PCa is graded depending on its aggressivity from 1 (least aggressive) to 5 (highest aggressivity) [3]. Based on these gradings, the European Association of Urology (EAU) further divides PCa into clinically insignificant (ciPCa), represented by ISUP 1 cases, and clinically significant PCa (csPCa), defined as having an ISUP of 2 or higher [4].

Multiparametric magnetic resonance imaging (mpMRI) holds a central role in terms of PCa diagnosis and staging [5], each lesion being assessed in accordance with the Prostate Imaging Reporting and Data System (PI-RADS) criteria [6]. Current guidelines recommend performing a prostate biopsy on all patients with a PI-RADS score ≥ 3 in order to confirm the potential malignancy [7]. However, the rate of detecting ciPCa is 42.8%; hence, there arises the need for additional filters that stratify the indication of invasive procedures [8].

The past decade has been marked by the advance of artificial intelligence (AI), with important applicability in medical specialties like urology, oncology, and radiology [9]. One of the most noteworthy applications of AI in the non-invasive diagnostic field is represented by texture analysis, a domain that quantifies statistically the spatial interrelation of pixels from a given region of interest, offering a mathematical equivalent to concepts such as rugosity, shape, and intensity [10]. This may apply to images sampled from digitally scanned pathology slides (tissue samples, cytology smears) or from routine imagistic investigations [11]. Taking the latter into consideration, radiomics is a domain dedicated to extracting metainformation from standard radiological acquisitions, such as the texture and pattern of pixels from regions of interest [12]. The implications would be the possibility of identifying csPCa solely on imagistic techniques, eluding the need for prostate biopsy in nearly half of the patients [13].

In order for radiomics to be fully integrated into the routine diagnostic workflow, automatic characterization of selected nodules is mandatory. Taking into consideration the high volume of data mined from texture analysis, it has been suggested that trained machine-learning (ML) algorithms may reach a performant prediction accuracy [14]. Recent studies have reported such algorithms that correctly identified csPCa nodules on T2-weighted images (T2WIs) in 81.8% of cases. However, these results were obtained when the study design employed the prostate biopsy pathological report as ground truth. When compared to the whole radical prostatectomy specimen, the accuracy of AI-based algorithms drops to 64% [15].

The aim of this study is to develop a machine-learning automatic classifier that can differentiate between indolent and csPCa cases, based upon mpMRI T2WI-derived texture features of the prostatic nodules.

## 2. Materials and Methods

### 2.1. Patient and Clinical Data Selection

We included in the current study all patients diagnosed with prostate cancer in our department who underwent 3D laparoscopic radical prostatectomy between June of 2022 and December of 2023. We collected the data prospectively, but the data analysis was performed in a retrospective manner. The indication for radical prostatectomy was based upon each patient’s staging, risk stratification, and personal preference, according to the European Association of Urology (EAU) Guidelines [16].

Data of interest included age, prostate-specific antigen (PSA) value, PI-RADS score per individual prostatic lesion, type of surgical approach, global and per-prostatic-nodule ISUP grade, as well as pathological tumoral staging of the surgical specimen.

The protocol was elaborated in accordance with the Ethical Principles for Medical Research Involving Human Patients and approved by the local Ethics Committee (No. 245/09.07.2022, issued by the University of Medicine and Pharmacy “Iuliu Hațieganu”, Cluj-Napoca, Romania). Informed consent was obtained from all subjects involved in the study.

### 2.2. Preoperative MRI Acquisition Protocol

All mpMRIs were performed prior to the intervention on the same 1.5 Tesla MRI scanner (MAGNETOM Aera^TM^, Siemens Healthcare, Erlangen, Germany), with a 16-channel phased-array body coil. All cases were examined using a standardized acquisition protocol: T2 turbo spin-echo (TSE) sequences in 3 planes (sagittal, coronal, and oblique axial), T1 TSE in the axial plane, and diffusion-weighted imaging (DWI) at b values of 50, 400, 800, 1000, and 1500, from which the software automatically rendered the apparent diffusion coefficient (ADC) maps. Additionally, dynamic contrast-enhanced (DCE) images were acquired immediately after contrast agent administration (Gadovist^TM^ 1.0; Bayer Schering Pharma AG, Berlin, Germany; 0.1 mmol/kg). Each acquisition was set at 25–35 slices, with a resolution of 640 × 640 pixels.

The mpMRIs were reviewed by 2 radiologists, reaching a consensus regarding tumor location and PI-RADS score.

### 2.3. Axial T2-Weighted Image Segmentation

**Figure 1:**
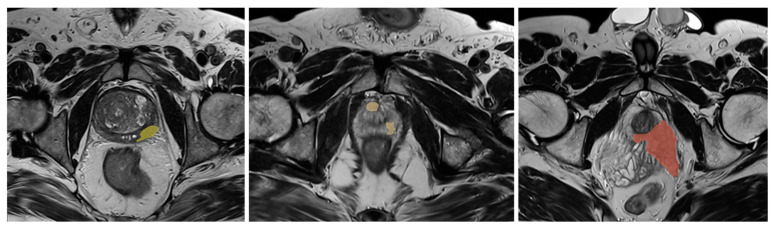
Dataset sample images, representing manually segmented T2WI images.

The oblique axial T2 TSE images were further processed using the 3D Slicer open-source software (version 5.2.2) [17]. Each nodule was manually delineated on each consecutive slide and analyzed individually. In cases with multiple lesions of interest per patient, each nodule was considered a stand-alone dataset (Figure 1).

**Figure 2:**
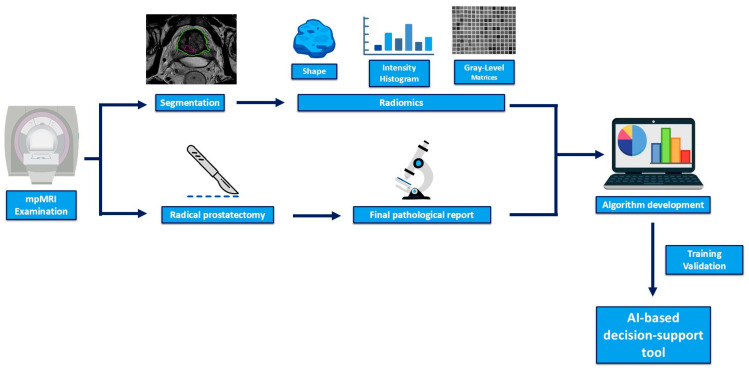
Graphical description of the study protocol.

Both radiologists were blinded to the final pathological report of the prostatectomy specimens. The segmentations were used to extract textural features, those proven to be statistically representative for each ISUP grade being further used to train the machine-learning algorithm for recognizing the characteristic patterns of the distinct ISUP categories. The study protocol and workflow are represented in Figure 2.

**Figure 3:**
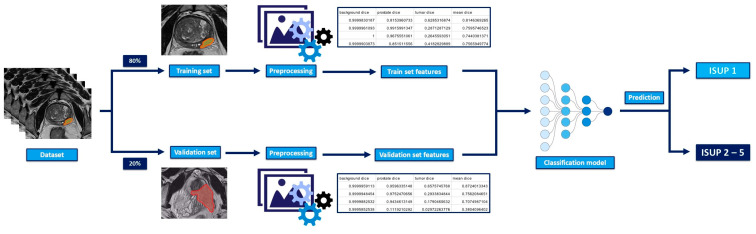
Graphical representation of the classification algorithm.

A detailed overview of the machine-learning classification algorithm is represented in Figure 3.

### 2.4. Surgical Approach

Radical prostatectomy was performed within 3 months of the initial mpMRI examination and subsequent prostate biopsy, either by 3D properitoneal laparoscopic approach using the Karl Storz 3D camera and laparoscopy cart (Tuttlingen, Germany) or by robotic transperitoneal approach using the Da Vinci X^TM^ surgical platform (Intuitive Surgical, Sunnyvale, CA, USA). The choice was at patients’ discretion. Both laparoscopic and robotic procedures were performed by the same urologist, with over 15 years of experience in minimally invasive surgery.

### 2.5. Pathology

The pathology assessment of the biopsies and radical prostatectomy specimens was performed by the same pathologist, with over 15 years of experience in uro-oncology. The surgical specimen was measured and processed entirely. The microscopical diagnosis was rendered on representative tissue samples of 3 μm thickness in standard hematoxylin-eosin stain. Immunohistochemical staining was used at the pathologist’s discretion. The overall and per-nodule ISUP grade was calculated according to the International Society of Urological Pathology Guidelines [18]. The staging was carried out according to the 2022 revision of the World Health Organization (WHO) classification of tumors of the urinary system and male genital organs [19].

### 2.6. Software Development

The algorithm was computed and rendered on a Windows-based laptop (Microsoft Corporation, Redmond, WA, USA), with the following configuration:

- Processor: 12th Gen Intel^®^ Core™ i7-1260P (Intel Corporation, Santa Clara, CA, USA);
- Central Processing Unit (CPU): 2.10 GHz;
- Random-Access Memory (RAM): 16 GB;
- Graphics Processing Unit (GPU): Intel^®^ Iris^®^ Xe Graphics;
- System Type: 64-bit operating system on an x64-based processor.

The manual segmentation datasets of each patient were converted into the NRRD (Nearly Raw Raster Data) (.nrrd) format. We rendered the 2D segmentations into a 3D Numpy array, using both renderings and individual 2D slices for further analysis. The images were subsequently processed by normalizing their intensity range, resampling their resolution scale (interpolation), and applying a discretization of the gray level matrices. Normalization with a scale of 100 was introduced in order to fit the intensities of images within the same range. Because texture calculation assumed isotropic spacing, we brought the images to the same resolution (scale) using the resampling (interpolation) function. To accommodate the slice thickness (~4 mm), while also avoiding generating too many interpolated voxels, we performed per-slice texture analysis with resampling set at 0.3 mm. Discretization with a bin width of 5 was applied to reduce image intensity to a set of discrete bins, which aided in robust feature extraction by minimizing the impact of noise. The classification algorithm was run on first-order features, derived from the intensity histogram, shape features, and textural features derived from the gray level co-occurrence matrix (GLCM), gray level run length matrix (GLRLM), gray level size zone matrix (GLSZM), gray level dependence matrix (GLDM), and neighboring gray tone difference matrix (NGTDM) [20,21,22]. For data processing, manually annotated masks were used. The PyRadiomics library (version 3.0.1) [23] was used to extract the features for manually contoured regions of interest within the prostate. Since we performed a per-slice feature computation, we used 2D shape features, as computed by the PyRadiomics library:

- Mesh Surface—The surface area of the 2D region, approximated based on the triangulated mesh of the boundary;
- Pixel Surface—The total surface area of the region based on the pixel count within the ROI;
- Perimeter—The length of the boundary of the region of interest in the 2D plane;
- Perimeter-to-Surface ratio—A measure of the compactness of the shape, calculated as the ratio of the perimeter to the surface area of the region;
- Sphericity—A measure of how closely the shape of the region approximates a perfect circle;
- Spherical Disproportion—A measure of the deviation of the shape from a perfect sphere, emphasizing irregularities in the region’s geometry;
- Maximum 2D diameter—The longest straight-line distance between any two points on the boundary of the region in the 2D plane;
- Major Axis Length—The longest axis of an ellipse fitted to the shape of the region, representing its primary direction of elongation;
- Minor Axis Length—The shortest axis of an ellipse fitted to the shape of the region, perpendicular to the major axis;
- Elongation—A measure of the extent to which the shape is stretched along its major axis relative to the minor axis.

Similarly to our previously published protocols, around 100 features were calculated per region [24].

For the classifier, we used the following machine-learning models: Random Forest, Balanced Random Forest (specially designed to be used for unbalanced datasets), and Support Vector Machine (SVM). This process involved identifying the most relevant features for building a classification model, thereby enhancing its accuracy and interpretability. By reducing the number of features and eliminating those deemed unnecessary or redundant, Random Forest was considered the best fit for constructing an effective model due to its inherent feature selection during the training process using Mean Decrease in Impurity (MDI) and permutation importance.

For the Random Forest model, we used scikit-learn’s implementation with the following parameters:

- Number of Trees: 100;
- Criterion: Gini index;
- Maximum Depth: None;
- Minimum Sample Split: 2;
- Minimum Sample Leaf: 1;
- Maximum Features: Auto;
- Bootstrap: True;
- Maximum samples: None;
- Class weight: Balanced.

Moreover, we introduced a cross-validation mechanism in order to provide a more reliable estimate of the algorithm’s performance than a single train–test split. To mitigate the imbalance between our classes, Stratified K-Fold was chosen due to its ability to ensure that each fold retains the same distribution of classes as the overall dataset. We generated 5 different train–test splits, each containing 80% training data and 20% test data, while maintaining the class distribution uniform. The final evaluation metrics were obtained by averaging the corresponding metrics for each individual train–test split. No data augmentation or oversampling strategies were employed.

### 2.7. Statistical Analysis

Using permutation importance, as provided by scikit-learn library, we extracted the top 30 most important features to our Random Forest model (Table 1). Both MDI and permutation importance yielded similar results in terms of identifying the top 30 features, although with slight differences in their ranking. Therefore, we opted to further employ the features retrieved by permutation importance methods, as they are considered to be less biased towards features’ cardinality. Additionally, for each of these subsets we performed the Student’s *t*-test and obtained its corresponding *p*-value. The t-test assumed the two groups had equal variances (for an independent sample *t*-test) and calculated the likelihood (*p*-value) that any observed difference between group means occurred by chance. A *p*-value below 0.05 indicated statistical significance. Features with a *p*-value below 0.05 exhibit statistically significant differences between the two classes (“ISUP 1” and “ISUP 2–5”).

To evaluate the performance of our model, we introduced four confusion matrix-based evaluation metrics: accuracy, precision, recall, and F1 score. These metrics were computed in a global manner and individually for each class (represented by *i* in the formulas below):

$$\mathrm{Precision}(i)=\frac{True\;positives(i)}{True\;positives(i)+False\;positives(1)}$$

$$\mathrm{Recall}(i)=\frac{True\;positives(i)}{True\;positives(i)+False\;negatives(i)}$$

$$F1(i)=\frac{2\times Precision(i)\times Recall(i)}{Precision(i)+Recall(i)}$$

The true positive, true negative, false positive, and false negative values were derived using confusion matrices. After computing the metrics for each class, we assessed the precision and recall of our model globally by applying 2 types of averaging on the per-class metrics:

- Micro-averaging, which calculates the aggregate metrics globally across all classes by treating all instances (individual predictions) equally (N representing the number of classes). It was deemed useful in terms of evaluating the overall performance of the classifier across all instances, giving equal weight to each instance.
  $$\mathrm{Micro-Precision}=\frac{\sum_{i=1}^{N}True\;positives(i)}{\sum_{i=1}^{N}[True\;positives\left(i\right)+False\;positives\left(i\right)]}$$
  $$\mathrm{Micro-Recall}=\frac{\sum_{i=1}^{N}True\;positives(i)}{\sum_{i=1}^{N}[True\;positives\left(i\right)+False\;negatives\left(i\right)]}$$
  $$\mathrm{Micro}-F1=\frac{2\times Micro-Precision\times Micro-Recall}{Micro-Precision+Micro-Recall}$$
- Macro-averaging, where each class of metrics was computed independently and then the average was taken across all classes. Each class was treated equally, regardless of its size or the number of instances it comprised (N representing the number of classes). It was deemed useful for understanding how the classifier performs on each class individually, especially when aiming to ensure that the classifier performs well across all classes, regardless of their frequency.

$$\mathrm{Macro-Precision}=\frac{1}{N}\sum_{i=1}^{N}Precision\;(i)$$

$$\mathrm{Macro-Recall}=\frac{1}{N}\sum_{i=1}^{N}Recall\;(i)$$

$$\mathrm{Macro}\text{-}F1=\frac{1}{N}\sum_{i=1}^{N}F1\;(i)$$

## 3. Results

### 3.1. General Characteristics of the Study Group

The protocol included 55 patients, summarizing a total of 76 prostate lesions. The median age of the study population was 64 years, with an interquartile range (IQR) of 61–68. The mean PSA value was 11.14 ng/mL, ranging between 3.5 ng/mL and 70 ng/mL. A total of 85.52% of the identified lesions had a PI-RADS score of 4 or higher. The pathological report of the radical prostatectomy specimens revealed organ-confined disease and clinically significant PCa in 80% and 85.45% of cases, respectively. The general characteristics of the study group are presented in Table 2.

### 3.2. Single Train–Train Split Versus Cross-Validation Model

Initially, we analyzed and compared the performance of a single train–test split model versus a five train–test split cross-validation algorithm. The results are illustrated in Table 3 and Table 4.

### 3.3. Performance of the Proposed Algorithm

After choosing the five train–test split cross-validation architecture, the performance of the algorithm was tested using all three classification models. The parameters for each are found in Table 5, Table 6 and Table 7.

Overall, the Random Forest machine-learning algorithm reached the highest accuracy in terms of distinguishing clinically insignificant cases from csPCas (87.2%).

When further dividing the study group into the four available ISUP scores, the accuracy of discriminating between each score based on the extracted textural parameters was 80.3% (Table 8).

Considering that every false negative for one class is another class’s false positive, Micro-Precision was equal to Micro-Recall. In this case, Micro-F1 was also equal to Micro-Precision and Micro-Recall. Furthermore, considering that every true negative for one class is another class’s true positive, accuracy proved to be equal to all three micro-averaged metrics.

## 4. Discussion

Prostate cancer is one of the pathologies at the forefront of healthcare strategies, with USD 9.8 billion being spent yearly on curative and palliative treatments [25]. Currently, prostate biopsy is recommended by the main guidelines as the accepted method for rendering the final PCa diagnosis, the opinions about non-invasive substitutes being still heterogeneous. However, it is a procedure that harbors several risks, such as hematuria (35.5%), hemospermia (26.3%), urinary retention (3.4%), and sepsis (3%) [26], as well as anxiety (64%) [27] and short-term erectile dysfunction [28]. By adding decision-support tools as additional steps of the routine diagnostic workflow, imagistic non-invasive surrogates can partake in the csPCa screening process, thus having the potential to replace prostate biopsies in active surveillance protocols.

The distinction between ISUP 1 and ISUP 2 or higher is important for determining aggressiveness and planning appropriate treatment strategies. Generally, lower ISUP grades are associated with less aggressive tumors that have a lower likelihood of developing locally advanced disease; thus, the aforementioned patients may become candidates for active surveillance. This, however, requires multiple prostate biopsies in order to assess a possible upgrading of the disease and, subsequently, a future upscale of therapy [29]. Therefore, by integrating decision-support tools that allow the automatic characterization of prostatic nodules, patients can avoid repeated invasive procedures and their inherent risks. Additionally, they end up benefiting from the global analysis of the nodule that, unlike biopsy cores that are limited to the sampled fragment, may give an overview of the condition from the first assessment, thus ensuring the shortest waiting time for receiving the correct treatment protocol. As proven by Nketiah et al. [30], integrating AI-based decision-support systems in the diagnostic workflow can increase the csPCa detection rate from 56% to 84%.

Our proposed approach for elaborating a computer-assisted diagnostic tool was the machine-learning type of algorithm, despite the emerging trend of deep-learning (DL) models. The main difference between the two AI methods resides in the need for manual training of the ML prototypes, as well as the data volume needed in order for them to complete the targeted task independently, such as differentiating between ISUP grades [31]. Unlike DL, ML requires much smaller datasets for training, thus representing a suitable choice for our study design of 76 samples and only one mpMRI acquisition used for segmentation. A systematic review published by Cuocolo et al. [32] reported an average sensitivity and specificity of 56% and 97%, respectively, as well as an accuracy of ML algorithms for detecting csPCa of 60–80%. Most referenced studies employed at least two mpMRI acquisitions, such as T2WI and ADC. In terms of DL models, Cai et al. [33] reached an accuracy similar to ours, of 89%, for differentiating csPCa. However, their algorithm was trained on 1454 datasets from three distinct mpMRI sequences (T1WI, T2WI, and ADC maps). Taking into consideration the heterogeneity of the studies in terms of algorithm architecture and employed mpMRI acquisitions, as well as the lack of data availability to assure their reproducibility, it is difficult to appreciate their real-life performance and to derive a cut-off value for the accepted diagnostic accuracy. Future standardized, multicenter, and multivendor trials are still needed in this emerging field.

Regarding our study protocol, one of the aspects that need to be addressed is the apparent class imbalance between the ISUP 1 and ISUP 2–5 groups. We addressed this imbalance by experimenting with classification models that are effective in reducing the bias toward the majority class and improving classification performance on the minority one, such as the Random Forest model. While data augmentation techniques, oversampling, and the Synthetic Minority Oversampling Technique (SMOTE) could have been used to address the class imbalance, we chose not to use them due to the risk of overfitting and of noise introduction, especially if the transformations could not have captured real-world variation effectively. To understand the impact, we examined the classification metrics for each class separately, and the model’s recall for ISUP 1 cases (a minority class) indeed proved to be low. This analysis shows that while the Random Forest model helps address class imbalance, there remains an inherent difficulty in achieving perfectly balanced predictive power due to the limited sample size of ISUP 1. However, since ISUP 2–5 represents clinically significant cancer with potentially distinct treatment or monitoring implications, the model’s performance on this class is particularly critical. Hence, in our evaluation and model selection, we prioritized approaches that enhance recall for ISUP 2–5 cases, ensuring these cases are correctly classified even if it involves a trade-off in overall accuracy or precision for other classes.

Another particularity of the proposed study is that we focused solely on T2WI acquisitions. While the current trend is to add supplementary sequences to the mpMRI protocols to increase their diagnostic accuracy, each addition represents an increase in the time needed per examination, leading to fewer patients scanned daily and an overcrowding of the waiting lists, with possible implications in delays of diagnosis. Concretely, a full protocol for an mpMRI of the prostate can take up to 45 min, out of which the T2-weighted images are obtained in 8–9 min [34]. Although not of diagnostic value alone, by having additional, automated decision-support tools that can characterize the suspect nodules, T2WI scans with incorporated ML algorithms may represent future screening tools that allow the evaluation of a large number of patients daily while maintaining acceptable accuracy. For this reason, we aimed to train the algorithm on T2WI alone to evaluate the maximum potential accuracy of a single-acquisition, ML-aided diagnostic device. However, further multicenter and multivendor studies are required to support the implementation of such screening modalities, as even the same acquisition protocol and parameter settings, when applied to two different MRI scanners, can produce subtle noise interferences that will affect the final diagnostic accuracy. Throughout the literature, studies that trained their in-house-built algorithms on acquisitions from one center or vendor and further tested it on external sources have reported drops in the validation accuracy of 15% to 25% [9,35], thus highlighting the need for larger, more varied datasets in the initial training subdivision.

To date, few papers have reported using texture analysis features extracted from only one mpMRI sequence, the preferred ones being those with the highest contrast of the tumoral tissue versus the background gland, such as diffusion or contrast-enhanced scans. Han et al. [36] employed textural features from ADC maps alone, reaching a sensitivity of 71.4%, a specificity of 80%, and an accuracy of 79.9% in terms of predicting high-grade PCas. Similarly, Winkel et al. [37] and Parra et al. [38] used DCE-based models, reporting sensitivities of 71% and 81%, specificities of 89% and 83%, and accuracies in detected csPCas of 90% and 82%, respectively. It is worth mentioning that all these studies focused on machine-learning algorithms. When it comes to the exclusive use of T2WI acquisitions, the literature is scant. Khosravi et al. [39] developed a DL-based protocol that was trained and validated on 400 T2WI in-house and publicly available datasets, reaching an accuracy of 89% for differentiating between benign and malignant tissues and 78% when differentiating low- and high-grade PCas. Likewise, Hectors et al. [35] published an ML-based model, trained on 212 benign prostatic lesions and 28 malignant ones, that reached an overall sensitivity and specificity of 75% and 79.8%, respectively, as well as an accuracy in terms of detecting csPCa on PI-RADS 3 mpMRIs of 76% in the training and 62% in the validation setting. Although focused on only one PI-RADS score, the authors acknowledge that the two classes in the study are highly unbalanced, thus cautioning about the need for further, more evenly sampled studies.

Taking all of the above into consideration, the particularity of our study protocol is the employment of a machine-learning architecture, trained on only one MRI acquisition (T2WI), that reached comparable accuracy to the more complex models cited in the current literature.

## 5. Limitations

Although the database was prospective, the analysis was performed retrospectively. Our study was based on a limited number of clinical datasets provided by a single tertiary-care center, all scans having been performed on the same MRI machine. This may result in lower csPCa detection accuracy than if the algorithm were validated on external or publicly available datasets. Therefore, further prospective, multicenter, and multivendor validation of the results is warranted.

## 6. Conclusions

To summarize, we developed an AI-based algorithm that proved to accurately differentiate between clinically significant and indolent prostate cancer in 87.2% of cases. By using exclusively T2-weighted acquisitions and robust machine-learning architecture, the proposed model has the potential of being easily integrated into the routine prostate cancer screening and diagnosis workflow, thus representing a valid option in terms of decision-support tools for computer-assisted diagnosis. However, these findings need to be extrapolated with caution, as they were obtained from a single-center, single-vendor research protocol. Future multicenter, multivendor, and multi-acquisition studies are needed before implementing the proposed decision-support tool on a larger scale.

## Figures and Tables

**Table 1:** The textural features extracted and used in the training of the machine-learning algorithm. GLDM = Gray level dependence matrix; GLCM = Gray level co-occurrence matrix; GLRLM = Gray level run length matrix; GLSZM = Gray level size zone matrix; Idn = Inverse difference normalized; Imc2 = Informational measure of correlation 2.

Feature	ISUP 1 Mean ± Standard Deviation	ISUP 2–5 Mean ± Standard Deviation	*T*-Test *p*-Value
Firstorder_RobustMeanAbsoluteDeviation	8.439 ± 3.114	11.041 ± 2.945	<0.001
GLDM_SmallDependenceHighGrayLevelEmphasis	62.393 ± 64.922	96.686 ± 62.104	0.012
Shape_Sphericity	0.833 ± 0.063	0.768 ± 0.087	<0.001
Firstorder_InterquartileRange	20.434 ± 7.597	26.485 ± 7.237	<0.001
Firstorder_MeanAbsoluteDeviation	12.893 ± 4.582	16.508 ± 4.360	<0.001
GLSZM_SizeZoneNonUniformity	42.036 ± 25.514	191.570 ± 243.407	0.003
GLCM_Idn	0.913 ± 0.023	0.929 ± 0.026	0.005
GLRLM_GrayLevelNonUniformityNormalized	0.102 ± 0.035	0.076 ± 0.018	<0.001
GLCM_ClusterTendency	38.125 ± 26.672	62.779 ± 37.859	0.002
Firstorder_Entropy	3.554 ± 0.501	3.966 ± 0.363	<0.001
Firstorder_Kurtosis	4.441 ± 1.971	4.303 ± 1.860	0.733
GLCM_Imc2	0.896 ± 0.055	0.913 ± 0.042	0.073
GLCM_SumEntropy	4.235 ± 0.519	4.731 ± 0.409	<0.001
GLSZM_HighGrayLevelZoneEmphasis	127.200 ± 101.213	223.129 ± 135.519	<0.001
GLSZM_SmallAreaHighGrayLevelEmphasis	93.581 ± 83.650	158.826 ± 98.714	0.002
GLCM_JointEntropy	6.078 ± 0.806	6.834 ± 0.673	<0.001
GLCM_JointEnergy	0.023 ± 0.014	0.013 ± 0.006	<0.001
GLCM_JointAverage	9.497 ± 3.976	12.937 ± 4.194	<0.001
Shape_Elongation	0.611 ± 0.153	0.615 ± 0.152	0.902
GLCM_MaximumProbability	0.060 ± 0.036	0.037 ± 0.017	<0.001
GLSZM_LowGrayLevelZoneEmphasis	0.046 ± 0.033	0.022 ± 0.018	<0.001
GLSZM_SmallAreaEmphasis	0.647 ± 0.094	0.672 ± 0.066	0.111
GLDM_SmallDependenceLowGrayLevelEmphasis	0.017 ± 0.011	0.009 ± 0.008	<0.001
GLDM_DependenceNonUniformityNormalized	0.276 ± 0.066	0.268 ± 0.062	0.582
GLRLM_LongRunHighGrayLevelEmphasis	188.402 ± 128.379	356.019 ± 245.142	0.001
GLCM_ClusterShade	113.160 ± 191.459	280.918 ± 729.566	0.264
Firstorder_90Percentile	0.969 ± 37.46	7.327 ± 28.060	0.318
GLDM_LowGrayLevelEmphasis	0.039 ± 0.028	0.019 ± 0.018	<0.001
GLDM_LargeDependenceLowGrayLevelEmphasis	0.233 ± 0.188	0.119 ± 0.158	0.001
GLRLM_RunLengthNonUniformity	128.450 ± 70.358	591.600 ± 795.223	0.004

**Table 2:** General characteristics of the patients enrolled in our study. Quantitative data are given as mean [range]. Qualitative data are given as numbers.

Variable	Value
Age (years)	65 [61–68]
PSA value (ng/mL)	11.14 [3.5–70.0]
Prostatic nodules	76
PI-RADS Score	
3	11
4	40
5	25
Radical prostatectomy approach	
LRP	69
RALP	7
pT staging per patient	
pT2	44
pT3	11
ISUP Grade per nodule	
Grade 1	14
Grade 2	49
Grade 3	10
Grade 4	0
Grade 5	3

**Table 3:** The accuracy of distinguishing clinically insignificant cases from csPCas using a single train–test split.

	PRECISION	RECALL	F1	ACCURACY
ISUP 1	0.5	0.25	0.333	0.777
ISUP 2–5	0.812	0.928	0.866	
MICRO-AVERAGING	0.777	0.777	0.777	
MACRO-AVERAGING	0.656	0.589	0.6	

**Table 4:** The accuracy of distinguishing clinically insignificant cases from csPCas using Stratified K-Fold cross-validation.

	PRECISION	RECALL	F1	ACCURACY
ISUP 1	0.6	0.2	0.293	0.822
ISUP 2–5	0.815	1	0.897	
MICRO-AVERAGING	0.822	0.822	0.822	
MACRO-AVERAGING	0.707	0.6	0.595	

**Table 5:** The accuracy of distinguishing clinically insignificant cases from csPCas using the Random Forest classification model.

	PRECISION	RECALL	F1	ACCURACY
ISUP 1	0.666	0.466	0.526	0.872
ISUP 2–5	0.888	0.963	0.923	
MICRO-AVERAGING	0.872	0.872	0.872	
MACRO-AVERAGING	0.777	0.715	0.725	

**Table 6:** The accuracy of distinguishing clinically insignificant cases from csPCas using the Balanced Random Forest classification model.

	PRECISION	RECALL	F1	ACCURACY
ISUP 1	0.314	0.533	0.388	0.674
ISUP 2–5	0.868	0.698	0.768	
MICRO-AVERAGING	0.674	0.674	0.674	
MACRO-AVERAGING	0.591	0.615	0.578	

**Table 7:** The accuracy of distinguishing clinically insignificant cases from csPCas using the Support Vector Machine classification model.

	PRECISION	RECALL	F1	ACCURACY
ISUP 1	0	0	0	0.802
ISUP 2–5	0.802	1	0.890	
MICRO-AVERAGING	0.802	0.802	0.802	
MACRO-AVERAGING	0.401	0.5	0.445	

**Table 8:** The accuracy of distinguishing between each subset of ISUP scores.

	PRECISION	RECALL	F1	ACCURACY
ISUP 1	0.525	0.392	0.477	0.803
ISUP 2	0.857	0.881	0.868	
ISUP 3	0.758	0.691	0.712	
ISUP 5	0.843	0.916	0.868	
MICRO-AVERAGING	0.803	0.803	0.803	
MACRO-AVERAGING	0.746	0.720	0.724	

## Data Availability

The original contributions presented in this study are included in the article. Further inquiries can be directed to the corresponding author.
